# Effect of Drug–Polymer Interaction in Amorphous Solid Dispersion on the Physical Stability and Dissolution of Drugs: The Case of Alpha-Mangostin

**DOI:** 10.3390/polym15143034

**Published:** 2023-07-13

**Authors:** Arif Budiman, Neng Vera Nurani, Eli Laelasari, Muchtaridi Muchtaridi, Sriwidodo Sriwidodo, Diah Lia Aulifa

**Affiliations:** 1 Department of Pharmaceutics and Pharmaceutical Technology, Faculty of Pharmacy, Universitas Padjadjaran, Jl. Raya Bandung-Sumedang Km. 21, Bandung 45363, Indonesia; neng19001@mail.unpad.ac.id (N.V.N.); eli19001@mail.unpad.ac.id (E.L.); sriwidodo@unpad.ac.id (S.S.); 2 Department of Pharmaceutical Analysis and Medicinal Chemistry, Faculty of Pharmacy, Universitas Padjadjaran, Jl. Raya Bandung-Sumedang Km. 21, Bandung 45363, Indonesia; muchtaridi@unpad.ac.id (M.M.); diah.lia@unpad.ac.id (D.L.A.)

**Keywords:** alpha-mangostin, amorphous solid dispersion, hydrogen bond, physical stability, dissolution

## Abstract

Improving drug solubility is necessary for formulations of poorly water-soluble drugs, especially for oral administration. Amorphous solid dispersions (ASDs) are widely used in the pharmaceutical industry to improve the physical stability and solubility of drugs. Therefore, this study aims to characterize interaction between a drug and polymer in ASD, as well as evaluate the impact on the physical stability and dissolution of alpha-mangostin (AM). AM was used as a model of a poorly water-soluble drug, while polyvinylpyrrolidone (PVP) and eudragit were used as polymers. The amorphization of AM-eudragit and AM-PVP was confirmed as having a halo pattern with powder X-ray diffraction measurements and the absence of an AM melting peak in the differential scanning calorimetry (DSC) curve. The solubility of amorphous AM increased in the presence of either eudragit or PVP due to amorphization and interactions of AM-polymer. Furthermore, FT-IR spectroscopy and in silico studies revealed hydrogen bond interactions between the carbonyl group of AM and the proton of eudragit as well as PVP. AM-eudragit with a ratio of 1:1 recrystallized after 7 days of storage at 25 °C and 90% RH, while the AM-PVP 1:4 and 1:10 samples retained the X-ray halo patterns, even under humid conditions. In a dissolution test, the presence of polymer in ASD significantly improved the dissolution profile due to the intermolecular interaction of AM-polymer. AM-eudragit 1:4 maintained AM supersaturation for a longer time compared to the 1:1 sample. However, a high supersaturation was not achieved in AM-PVP 1:10 due to the formation of large agglomerations, leading to a slow dissolution rate. Based on the results, interaction of AM-polymer in ASD can significantly improve the pharmaceutical properties of AM including the physical stability and dissolution.

## 1. Introduction

Poorly water-soluble drug substances are growing commercially in drug product development but are associated with insufficient bioavailability through oral administration [1,2,3,4]. Over 40–70% of recently developed new drug candidates and marketed products are poorly soluble in water based on the Biopharmaceutical Classification System (BCS) [5]. The Developability Classification System (DCS) was also considered by formulators in the development of poorly soluble drug formulations to identify the root cause for low solubility and provide strategies for the drugs that are either dissolution-limited or solubility-limited. BCS Class II was divided into two sub-categories: DCS Class IIa (the drugs that have rate-limited dissolution) and DCS Class IIb (the drugs that have below the solubility-limited absorbable dose) [6]. This creates a difficult challenge in the development of oral dosage forms due to their associated low bioavailability [7,8]. Therefore, developing a strategy to improve drug solubility is necessary, especially for oral administration [9].

Drug amorphization is one of the most promising formulations for enhancing dissolution behavior and the bioavailability of poorly water-soluble drugs. Amorphous drugs tend to have disordered structures and higher free energy compared to their crystalline counterparts, which improves their aqueous solubility [10,11,12]. When dispersed in water, they form supersaturated solutions, leading to the improvement of oral bioavailability [13]. However, amorphous drugs are thermodynamically unstable and easily recrystallized either during storage or after being dispersed in water. This makes it practically difficult to use them alone in solid formulations. The addition of an excipient is needed to inhibit the recrystallization of amorphous drugs.

Amorphous solid dispersion (ASD) is a strategy to inhibit the recrystallization of amorphous drugs by dispersing them into a polymer matrix [7]. The intermolecular interactions [14,15,16] between a drug and polymer within ASD increase the dissolution rate and solubility of the drug and improve its physical stability [17]. In addition, the strong interactions lead to effective inhibition of recrystallization for amorphous drugs. This implies that selecting a suitable polymer as a carrier is crucial to convert the crystalline drug into its amorphous form and to stabilize ASD by reducing the molecular mobility and extending the glass transition temperature (*T_g_*) [18].

Although the formulation of ASD with various polymeric additives has been extensively investigated, the interaction mechanism of each drug would be different due to their unique physicochemical properties [19]. The mechanism of drug–polymer interaction and its impact on the pharmaceutical properties would also be different for each drug. This study was conducted to investigate the mechanism of interaction between alpha-mangostin (AM) and polymer based on a molecular-level characterization and its relationship with their pharmaceutical properties, such as dissolution profile and physical stability. AM was used as a model of a poorly water-soluble drug because it is a potentially active compound used as an antitumor treatment which has a low water solubility, leading to low oral bioavailability. Meanwhile, polyvinylpyrrolidone (PVP) [20,21] and eudragit were used as polymers because they have been widely utilized in ASD and showed good ability in drug crystallization inhibition either after storage or dispersion in water [22,23]. Lehmkemper et al. reported that the presence of PVP can improve the physical stability of amorphous naproxen and acetaminophen [20]. The stability of curcumin was also greatly enhanced with the presence of eudragit in amorphous solid dispersion [24]. Understanding the interaction mechanism between AM and each polymer as well as its impact on the pharmaceutical properties is necessary to make ASD formulations, specifically for oral administration.

## 2. Materials and Methods

### 2.1. Materials

**Figure 1:**
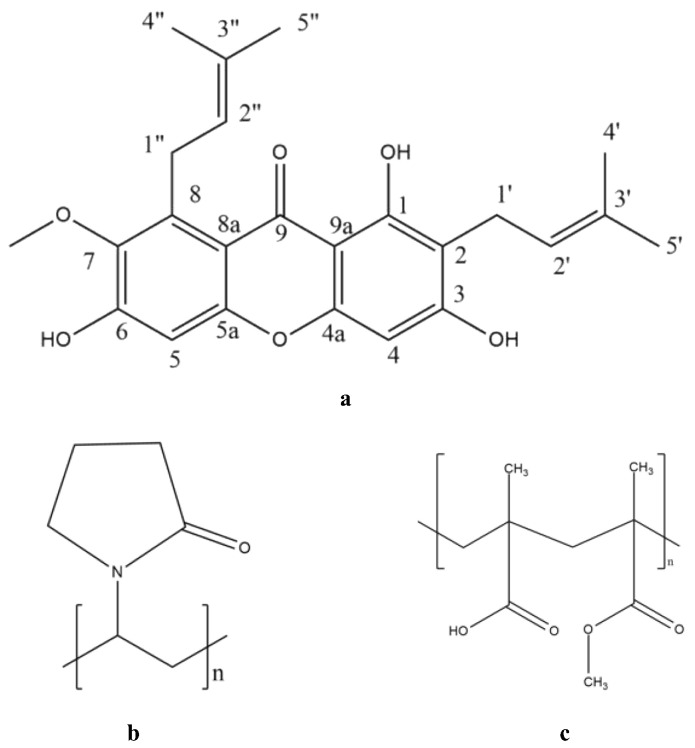
Chemical structures of (**a**) AM, (**b**) PVP, and (**c**) eudragit.

AM with a molecular weight of 410.5 g/mol was bought from Chengdu Biopurify Phytochemicals (Shincuan, China), while PVP and eudragit were purchased from Sigma-Aldrich (Singapore). The chemical structures of AM, PVP, as well as eudragit are presented in Figure 1, and all chemicals were used as received without further purification.

### 2.2. Preparation of Amorphous Solid Dispersion (ASD)

ASD was prepared using the solvent evaporation method by dissolving AM and each polymer (PVP and eudragit) in methanol in a ratio of 1:1. 1:4, 1:6, 1:8, and 1:10 (w/b). The solution was evaporated using a Buchi Rotavapor-R (Buchi Corp, New Castle, DE, USA) attached to a water bath (BM200, Yamato Scientific America, Santa Clara, CA, USA) kept at 30 °C. The residual powders were then dried at 30 °C for 72 hours using an oven to obtain the samples.

### 2.3. Powder X-ray Diffraction (PXRD) Measurement

PXRD patterns were obtained using a Kristalloflex diffractometer (Bruker D8 Advance) (Siemens, Berlin, Germany) with the following conditions: voltage 40 kV, target Cu, filter Ni, current 40 mA, scanning rate 1°/min, and scanning angle of 2θ = 10°–40°.

### 2.4. Differential Scanning Calorimetry (DSC) Measurement

The DSC profile of each sample was collected using a DSC device (Shimadzu DSC-60 plus, Kyoto, Japan). Approximately 3–5 mg of the samples was placed into a crimped aluminum DSC pan under a N2 purge at a flow rate of 20 mL/min. Samples were measured from 0 to 280 °C at a heating rate of 10 °C/min.

### 2.5. Fourier Transform Infrared (FT-IR) Spectroscopy

IR spectra of each sample were obtained using a Prestige21 IR spectrometer (Shimadzu, Kyoto, Japan) to determine the interaction between each polymer and AM. Samples of 1–2 mg were dispersed in 200–250 mg of potassium bromide powder. The mixture was homogenized with a mortar and pestle, then compressed into a pellet with a pressure of 60 Psi, while the spectra were recorded at wave numbers 4000–400 cm^−1^.

### 2.6. Crystalline and Amorphous Solubility Determination

The solubility of AM crystal and ASD prepared by solvent evaporation was determined using 50 mM phosphate buffer at pH 7.4 and 37 °C. The excess amount of each sample was dispersed into a solution of phosphate buffer at pH 7.4 and shaken for 48 h at 37 °C. Subsequently, the solutions were filtered through a 0.45 μm membrane filter, diluted with mobile phase, and assessed using high-performance liquid chromatography (HPLC). Amorphous solubility of AM was also determined using centrifugation, as described in a previous report by Dening et al. (2018) [25].

### 2.7. HPLC Conditions

HPLC analysis was carried out using a Dionex-Ultimate 3000 HPLC (Dionex, Sunnyvale, CA, USA). The sample was injected into an Inertsil ODS C18 (Torrance, California, USA) with a 6.0 × 150 mm column at 30 °C, while the composition of the mobile phase was acetonitrile and 0.1% formic acid in water at a ratio of 95:5. The samples were detected using a UV detector at a wavelength of 244 nm, and the standard solutions were prepared in the mobile phase with concentrations of 1, 5, 10, 25, 50, 100, and 200 μg/mL.

### 2.8. Entrapment Efficiency Measurement

The entrapment efficiency of ASD with various ratios was determined by adding each sample into methanol at an AM concentration of 100 μg/mL with stirring for 30 min. The samples were filtered through a 0.45 µm membrane filter, diluted with acetonitrile, and analyzed using HPLC.

% Entrapment efficiency = (measurement concentration/theoretical concentration) × 100(1)

### 2.9. Dissolution Experiment

Dissolution measurements were carried out using the paddle method wherein the powder samples were dispersed in 500 mL of 50 mM phosphate buffer with pH 7.4 as a dissolution medium at an AM concentration of 40 µg/mL. The solution was stirred at 37 °C with a 150 rpm rotating paddle. Subsequently, 5 milliliters of dissolution medium were withdrawn at intervals of 5, 10, 20, 30, 60, 90, 120, and 150 min. The samples were filtered through a 0.45 μm membrane filter, diluted with acetonitrile, and analyzed via HPLC.

### 2.10. Storage Stability Study

Physical stability tests were performed by storing each sample under different conditions at 25 °C and 0% relative humidity (RH) in a desiccator containing silica gel, as well as (b) 25 °C and 90% RH in a desiccator containing saturated potassium nitrate solution [26], then the samples were evaluated using PXRD after 30-day storage.

## 3. Results

### 3.1. PXRD Measurement

**Figure 2:**
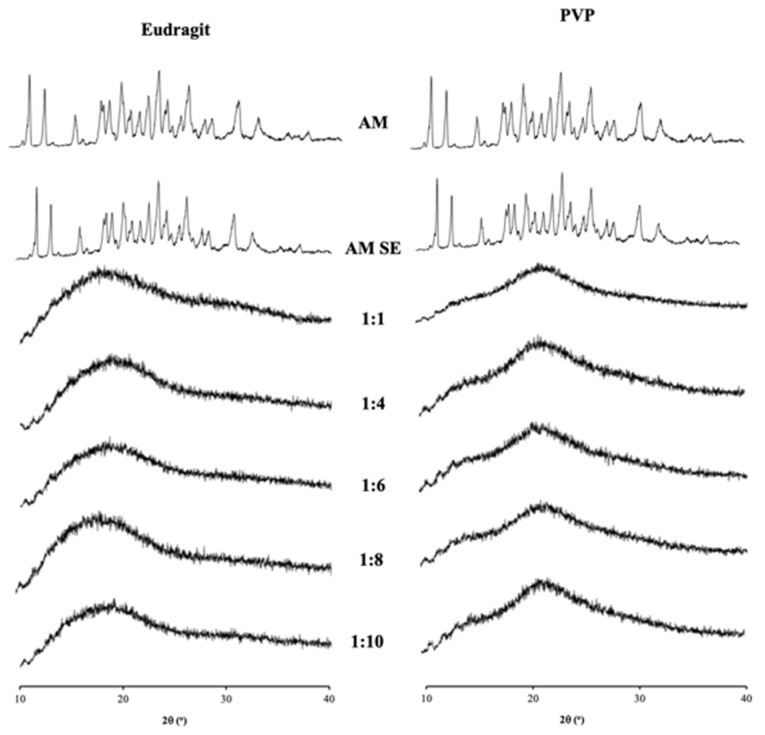
PXRD patterns of AM, AM SE, AM-eudragit, and AM-PVP with various weight ratios.

AM crystal showed characteristic diffraction peaks in PXRD patterns, while PVP and eudragit had a halo pattern, representing their amorphous state as shown in Figure 2. The characteristic diffraction peaks were also observed in the AM prepared by solvent evaporation (AM SE), indicating that amorphous AM was not formed due to its high recrystallization tendency. This result might be attributed to AM being classified as class I according to Taylor’s classification [27]. In contrast, AM-PVP and AM-eudragit showed a halo pattern without any diffraction peaks at all weight ratios, suggesting the formation of ASD. This indicates that amorphous formulations of AM-PVP and AM-eudragit can be prepared by the solvent evaporation method.

### 3.2. DSC Measurement

**Figure 3:**
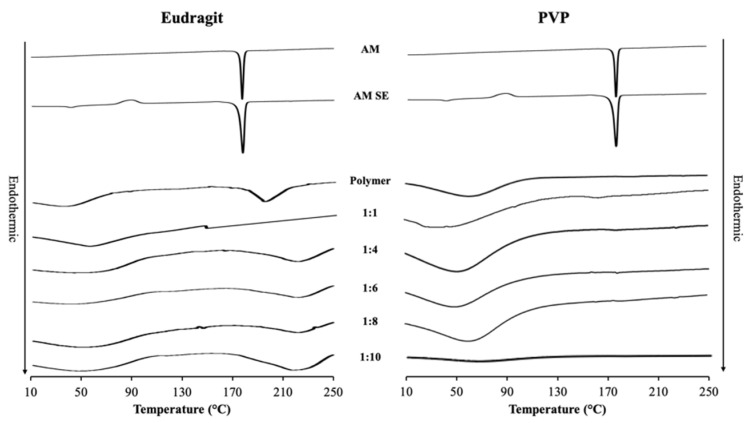
DSC curves of AM, AM SE, AM-eudragit, and AM-PVP with various weight ratios.

The crystal and AM SE showed an endothermic melting peak at around 177 °C in DSC profiles as shown in Figure 3, while the endothermic melting peaks of PVP and eudragit were not observed due to their amorphous state. The endothermic melting peak was also not observed in AM-PVP and AM-eudragit at all weight ratios even after heating, indicating that amorphous AM was formed in the presence of PVP as well as eudragit. Moreover, the DSC profile suggests that the amount of each polymer was sufficient to stabilize all of the amorphous AM even in the ratio of 1:1. The results of the PXRD pattern and DSC profile indicate that the formation of ASD occurred in AM-PVP and AM-eudragit prepared by the solvent evaporation method.

### 3.3. Entrapment Efficiency

**Figure 4:**
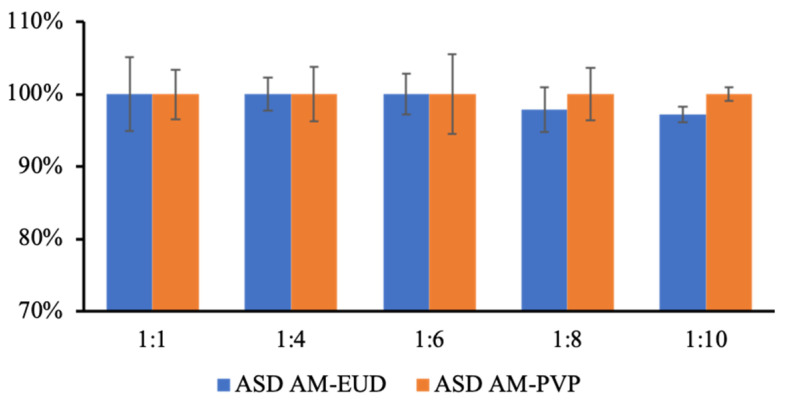
The entrapment efficiency of AM-eudragit, and AM-PVP with various weight ratios.

The entrapment efficiency of AM-PVP and eudragit was measured for all produced formulations by dispersing the sample into methanol. Since eudragit and PVP are not absorbed at approximately 244 nm, the presence of AUC in the HPLC measurement could be attributed to a function of AM concentration. Figure 4 shows that the entrapment efficiency of all produced formulations from each polymer was almost 100%. This result indicates that AM was effectively entrapped in PVP and eudragit, respectively, leading to a high concentration in the ASD system.

### 3.4. Solubility Measurement

The solubility was measured to characterize the relative hydrophobicity between the polymer and the model compounds. The equilibrium solubility of AM was determined using 50 mM phosphate buffer of pH 7.4 and a temperature of 37 °C. The crystalline AM equilibrium solubility value where the molecules exist in the un-ionized form was 0.43 ± 0.3 μg/mL, indicating that AM has extremely poor aqueous solubility. Furthermore, Table 1 shows that the solubility was improved in the presence of PVP as well as eudragit. In other words, the solubility of amorphous AM increased with a rising polymer concentration in ASD. A previous study reported that eudragit can increase the solubility of a drug through the solubilization effect [28]. The solubility increased because drug solubilization can reduce the solute thermodynamic activity, hence, amorphous solubility of a drug can be increased by adding solubilizing components such as polymer [29,30]. Meanwhile, the solubility enhancement from AM in the presence of PVP might be related to several mechanisms including complexation with PVP, changing the physical state from a crystalline to amorphous state, particle size reduction, and self-assembly into nanomicelles. It has also been reported that the AM-PVP complex generates the encapsulation of hydrophobic compounds in the core, leading to solubility enhancement of AM in aqueous media [31,32]. Nevertheless, amorphization could be the main reason for AM solubility enhancement, because a previous study reported that the presence of PVP and eudragit showed only slight effect on the thermal equilibrium solubility, especially at a concentration of above 2000 μg/mL [33].

### 3.5. FT-IR Spectroscopy Analysis

**Figure 5:**
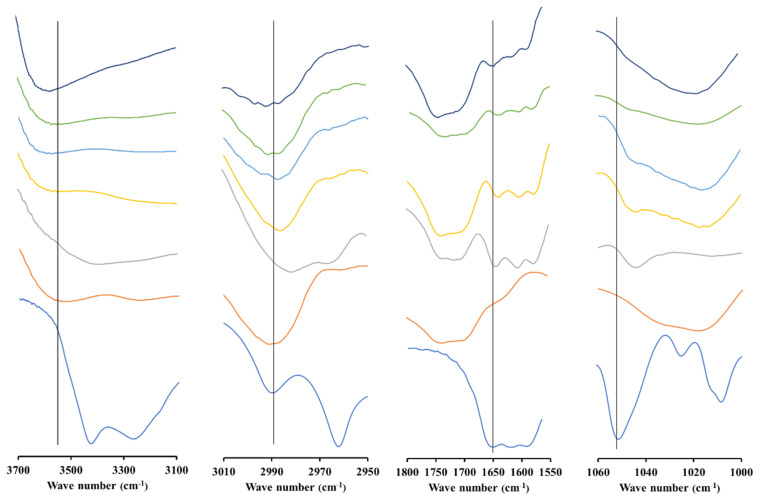
FT−IR spectra of AM−eudragit with various weight ratios. **ꟷ **AM, **ꟷ **Eudragit, **ꟷ **AM-Eud 1:1, **ꟷ**AM-Eud 1:4, **ꟷ**AM-Eud 1:6, **ꟷ**AM-Eud 1:8, **ꟷ**AM-Eud 1:10.

**Figure 6:**
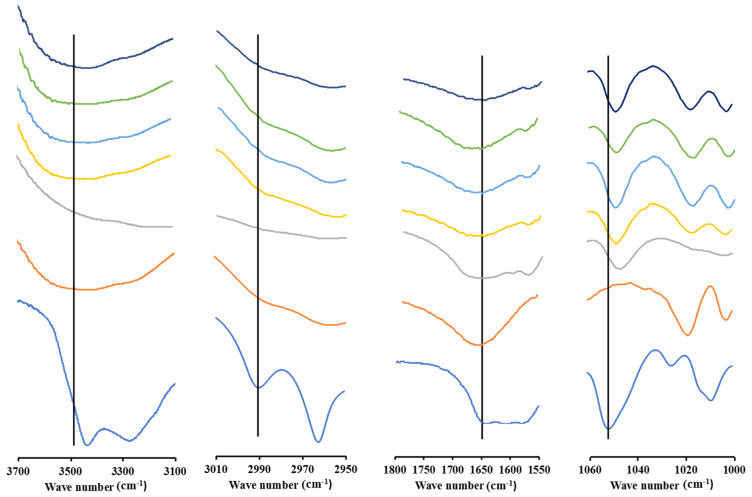
FT−IR spectra of AM−PVP with various weight ratios. **ꟷ **AM, **ꟷ **PVP, **ꟷ **AM-PVP 1:1, **ꟷ**AM-PVP 1:4, **ꟷ**AM-PVP 1:6, **ꟷ**AM-PVP 1:8, **ꟷ**AM-PVP 1:10.

FT-IR spectroscopy was used to confirm interactions formed between AM and polymer in the ASD system as shown in Figure 5 and Figure 6. The results demonstrate that AM showed spectra at wave numbers 3410 and 3280; 2995 and 2965; as well as 1642 and 1051 cm^−1^, which represent the OH, CH, C=O, and C-O stretching functional groups, respectively. ASD of AM-eudragit with various weight ratios exhibits a shift in the (C=O) carbonyl group from wave number 1642.41 cm^−1^ to around 1639 cm^−1^. The shift in spectra was also observed in the AM (C-O) group from 1051.22 cm^−1^ to around 1042 cm^−1^. This indicates that there is an intermolecular interaction between the carbonyl group of AM as a hydrogen acceptor and eudragit as a hydrogen donor occurring in ASD. In this system, the (C-H) functional group spectra also shifted from 2989,71 cm^−1^ to around 2985 cm^−1^ due to similar intermolecular interactions to those explained previously. The (O-H) functional group spectra also shifted from 3527.25 cm^−1^ to 3561 cm^−1^.

ASD AM-PVP IR spectrum analyses showed that the (C-H) functional group spectra shift from 2995 cm^−1^ to 2958 cm^−1^. The shift can be attributed to the possible intermolecular interaction between PVP hydrocarbon groups as hydrogen donors and AM as a hydrogen acceptor in the ASD AM-PVP spectrum. The carbonyl group (C=O) AM and PVP spectra can be observed at 1644.34 cm^−1^ and 1676.81 cm^−1^, respectively. The carbonyl group spectrum in AM-PVP shifted to around 1676 cm^−1^. The shift in spectra was also observed in the AM (C-O) group from 1086.91 cm^−1^ to 1082.08 cm^−1^. The shifts of hydroxyl groups (O-H) of AM and PVP were observed at wave numbers of 3420 cm^−1^ and 3480 cm^−1^, respectively. The shifts in the spectrum were due to the intermolecular interaction between AM as a proton acceptor and PVP as a proton donor.

### 3.6. In Silico Study

**Figure 7:**
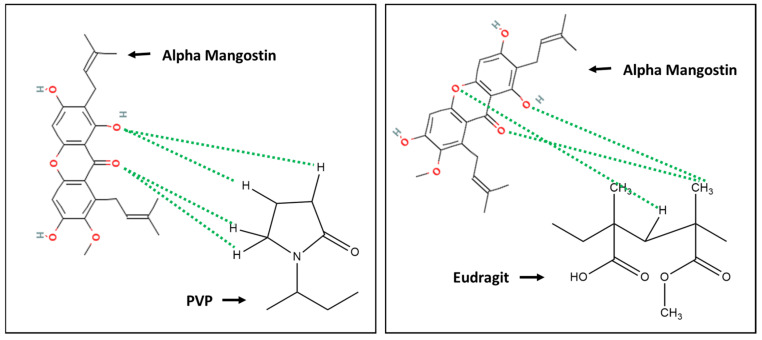
Two-dimensional visualization of AM with PVP and eudragit in ASD. Adapted from data presented originally in [33]. **(- - -**) hydrogen bond interaction. Reproduced with permission from Arif et al, (2022).

A previous study predicted interaction between AM-PVP and eudragit using the ligand–ligand method with the software Discovery Studio Visualizer and Autodock Tools, by considering the binding energy (Ei) and distance of each contact formed between AM and each polymer (Figure 7). The results showed that interaction of hydrogen bonds was observed between the carbonyl group of AM and the hydrocarbon group of PVP polymer. The interaction distance was 0.52 Å and 1.54 Å for PVP and eudragit, respectively, with a bond energy of −1.5 kcal/mol. Meanwhile, the formation of hydrogen bond interactions was observed between the carbonyl group of AM and the hydrocarbon group of eudragit with a distance value of 1.75 Å and a bond energy of −0.9 kcal/mol. These results show that intermolecular interaction between AM and PVP as well as eudragit in ASD is based on their energy values and bond distances.

### 3.7. Physical Stability Test

**Figure 8:**
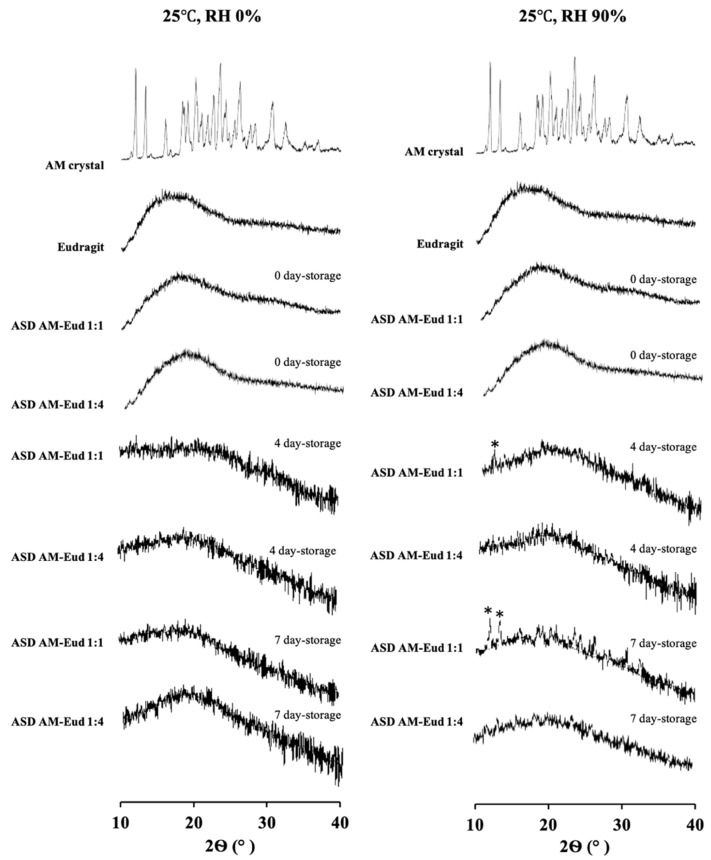
PXRD patterns of AM crystal, AM SE, AM-eudragit 1:1, and AM-eudragit 1:4 after storage at 25 °C 0% RH (**left**) and 40 °C, 90% RH (**right**). (*) Characteristic peaks of AM.

The physical stability of AM in AM-eudragit was monitored with PXRD measurements as shown in Figure 8. AM-eudragit 1:1 and 1:4 samples remained stable in a dry powder state after 7 days of storage at 25 °C. In contrast, the 1:1 sample recrystallized after 7 days of storage at 25 °C with RH of 90%. The humidified conditions induced recrystallization in AM-eudragit 1:1, while an amorphous state was maintained in AM-eudragit 1:4. This is because AM-eudragit 1:4 has lower wettability and stronger hydrogen bond interaction compared to the 1:1 sample. The *T_g_* values of AM in ASD were further lowered by water adsorption under humidified conditions, leading to recrystallization. Moreover, the sticky mass of AM in ASD occurred under humidified conditions due to the supercooled liquid state of the samples. AM-PVP 1:10 showed halo patterns even after 30 days of storage at 25 °C either with 0% or 75% RH as shown in Figure S1. The low wettability might be due to its self-assembly into nanomicelles which could prevent water from coming into contact with AM in eudragit.

### 3.8. In Vitro Dissolution Test

**Figure 9:**
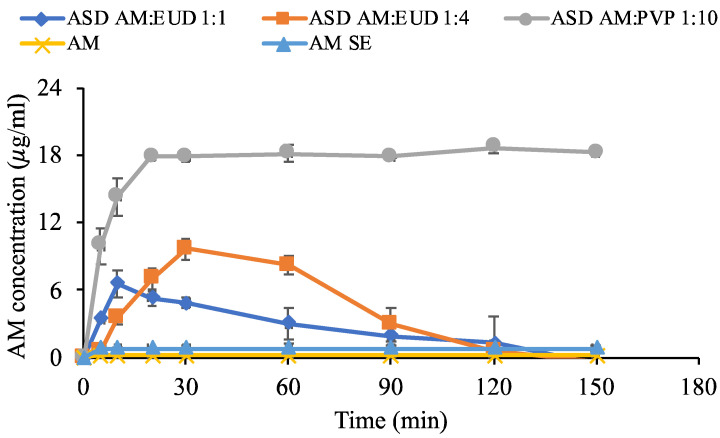
Dissolution profiles of each sample in 50 mM phosphate buffer (pH 7.4) at 37 °C (n = 3, mean ± SD).

A dissolution test of AM in ASD was performed in 50 mM phosphate buffer at pH 7.4 and 37 °C under non-sink conditions as described in Figure 9. AM crystal showed slow dissolution at a concentration of 0.18 μg/mL. Dissolution of AM SE was almost similar to that of AM crystal. This indicates that amorphous AM alone cannot be prepared by the solvent evaporation method due to its high recrystallization tendency. Dissolution of AM-eudragit 1:1 improved compared to AM crystal as well as AM SE because it showed a spring-parachute dissolution profile commonly observed for amorphous systems. Rapid release occurred at the beginning of dissolution, and the concentration of AM was found to be approximately 6.59 μg/mL. This concentration is almost similar to the amorphous solubility of AM-eudragit 1:1. This result indicates that AM-eudragit 1:1 has good dispersibility in dissolution medium, leading to the rapid dissolution of AM in ASD. However, the concentration gradually decreased due to recrystallization in dissolution medium. AM dissolution from AM-eudragit 1:4 was considerably different from that of 1:1. At the beginning of the test, dissolution in AM-eudragit 1:4 was rather slow compared to that of 1:1. This is presumably due to the presence of more polymer which could delay the release of AM, leading to the slower rate of dissolution. However, the concentration reached about 9.63 μg/mL at 30 min which was almost similar to the amorphous solubility of AM-eudragit 1:1. The recrystallization of AM in AM-eudragit 1:4 was slower compared to that of AM-eudragit 1:1 and these data are in agreement with the result of physical stability. This implies that interaction and incorporation of AM into the matrix of polymer could generate and maintain supersaturation. In contrast, the concentration of AM-eudragit 1:10 could not be detected even after 150 min, presumably due to its low wettability culminating in a slow dissolution rate. The concentration of AM in this sample was higher than that of AM-eudragit due to its amorphous solubility being higher than AM-eudragit 1:1 or 1:4. However, the high supersaturation of AM was not achieved in AM-PVP 1:10, and the concentration only reached half of its amorphous solubility even after 150 min. This result suggests that AM in AM-PVP 1:10 was not well dispersed in dissolution medium, forming large agglomerations and a slow dissolution rate. Similarly, the concentration of AM in AM-PVP 1:1 and AM-PVP 1:4 could not be detected even after 150 min. The poor wettability of both samples might have contributed to the low dissolution rate of AM but these phenomena need to be confirmed by additional experiments in future studies.

## 4. Discussion

This study examined the mechanism of interaction between AM and polymers in ASD as well as its impact on the physical stability and dissolution profile. Amorphous AM was formed after being prepared by solvent evaporation with the presence of either eudragit or PVP, while recrystallization was still observed in the sample without polymer. This suggests that interaction between AM and each polymer could reduce the mobility and nucleation rate of amorphous AM, thereby inhibiting recrystallization and increasing the physical stability in ASD. Interaction between AM, as well as PVP, and eudragit was confirmed by FT-IR spectroscopy. The results showed that the carbonyl group of AM interacted with the proton of eudragit as well as PVP, contributing to recrystallization inhibition in ASD.

The high solubility of amorphous AM is known and has been extensively reported in several previous studies [11,34,35]. This is attributed to the disordered structure of amorphous solid. In a crystalline state, the lattice crystal must be disrupted for the material to dissolve, while in an amorphous state, only short-range intermolecular interactions occur. The solubility of amorphous AM increased with higher amounts of either eudragit or PVP in ASD. When the polymer amount is low, the solubility improvement of AM is caused by amorphization. However, increasing the amount of polymer leads to not only the change in the physical state from crystalline to amorphous form but also the formation of porous solid dispersion, particle size reduction, and self-assembly into nanomicelles, which is in agreement with a previous study [36]. It should be noted that the solubility improvement of AM also differed depending on the type of polymer. Solubility evaluation with the presence of polymer showed that eudragit improved drug solubility better compared to PVP at all concentrations. This suggests that the ratio between drug and polymer in ASD and the choice of polymer are important factors in amorphous solubility of AM [37].

The physical stability of a drug is very important in the development of oral solid dosage because it is crucial to maintaining bioavailability in an ASD system. Meanwhile, the humidity condition has a strong influence on physical stability. The different polymers showed varying hydrophilicity, leading to variations in the water-sorption properties and the physical stability. AM-eudragit 1:1 recrystallized after 7 days of storage at 25 °C and RH of 90%, while the 1:4 sample retained its amorphous state. This is presumably because, in the 1:4 sample, AM was occupied or surrounded by polymer, which prevented contact with water and led to slower recrystallization compared to the 1:1 ratio. Meanwhile, AM-PVP 1:10 was stable even after 7 days of storage at 25 °C and RH of 90%. This is due to the successful formation of self-assembled nanomicelles, which embedded amorphous AM in polymer and suppressed the critical nucleus size, thereby inhibiting recrystallization [38]. AM-PVP was found to be more physically stable compared to AM-eudragit due to the difference in hydrophilicity and the water sorption of each polymer, leading to the variation in the glass transition temperature suppression and subsequent recrystallization inhibition.

**Figure 10:**
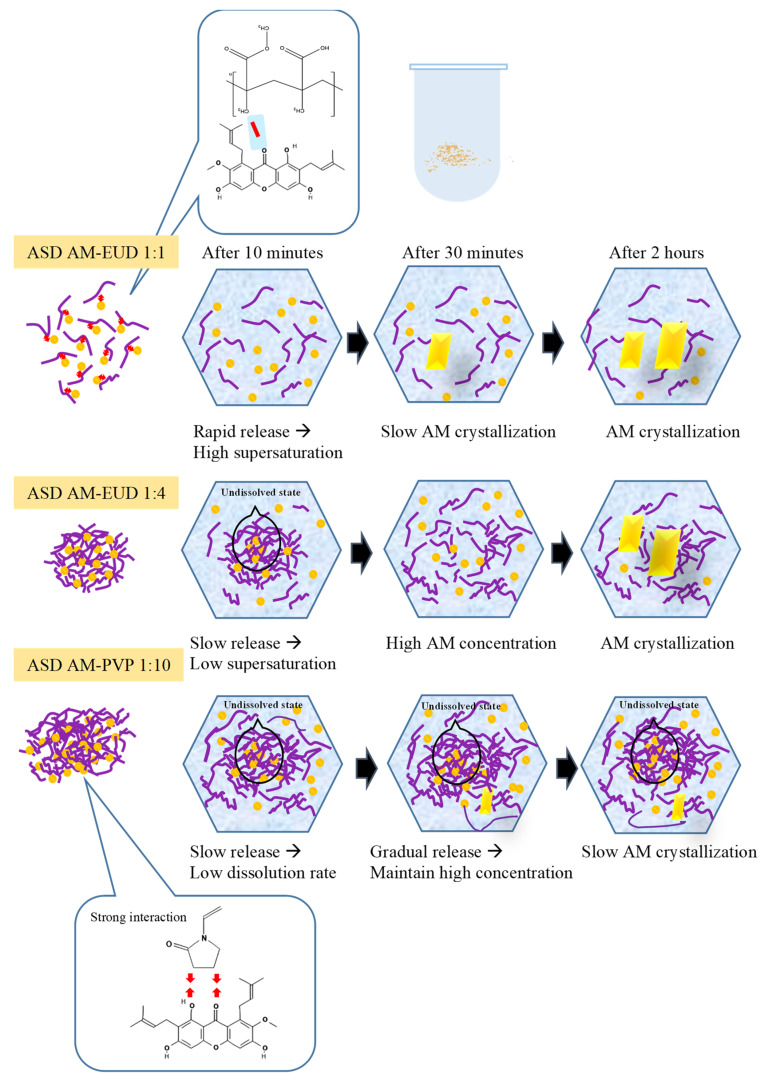
Schematic illustration of AM dissolution from AM-eudragit 1:1, AM-eudragit 1:4, and AM-PVP 1:10. (**-**) and (→ ←) is the hydrogen bond interaction.

The proposed mechanism of AM dissolution from AM-PVP and AM-eudragit in ASD is summarized in Figure 10. In AM-eudragit 1:1, the monomolecularly dispersed AM rapidly dissolved into dissolution medium through interaction of water molecules with the carbonyl group. AM was rapidly released from the polymer matrix into the bulk dissolution medium. A high supersaturation level or concentration of AM was achieved at the beginning of the dissolution test due to the rapid dissolution. However, the concentration gradually decreased over time due to the crystal growth in the bulk solution. In the case of AM-eudragit 1:4, the rapid release of AM at the beginning of the dissolution test was inhibited due to the suppression of water contact in ASD. The carbonyl group of AM was occupied by hydrogen bonding with the proton of eudragit, leading to a slower dissolution rate compared to AM-eudragit 1:1 sample. The gradual release from the matrix of polymer into dissolution medium inhibited nucleus formation and the subsequent recrystallization due to hydrogen bonds between AM and eudragit. Meanwhile, in the case of AM-PVP 1:10, the agglomeration of AM in ASD suppressed the surface area and caused low wettability. The water only interacted with the surface of amorphous AM after being dispersed into the medium and it gradually dissolved, leading to a low dissolution rate. A high supersaturation was also not achieved and the concentration remained unchanged even after being dispersed into the medium for 150 min, despite the AM concentration being higher than that of AM-eudragit. This phenomenon occurred because some of the AM was recrystallized in the bulk medium, while some was also dissolved gradually. This result was similar to our previous study in which the formation of agglomeration by the drugs decreased the surface area, leading to low wettability and dissolution rate of amorphous drugs [39]. Therefore, the concentration did not significantly change in dissolution medium for 150 min.

## 5. Conclusions

This study was conducted to investigate the physical stability and dissolution properties of amorphous systems consisting of AM-eudragit and AM-PVP. AM with a high recrystallization tendency was successfully amorphized in ASD with eudragit and PVP, respectively, through the solvent evaporation method. The amorphization occurred through hydrogen bonds formed between the carbonyl groups of AM and polymer protons. This interaction inhibited the recrystallization of AM after storage in humidified conditions. Moreover, hydrogen bond as well as nanomicelle formation helped to maintain AM supersaturation for a long time. This study, in general, provides fundamental insight into ASD formulation, especially for active compounds sourced from herbal plants which have a high recrystallization tendency.

## Figures and Tables

**Table 1:** Crystalline (S_c,AM_) and amorphous (S_a,AM_) solubility of AM in 50 mM phosphate buffer at pH 7.4.

Sample	S_c,AM_	S_a,AM_	S_a,AM_/S_c,AM_
AM SE	0.43 ± 0.3	0.44 ± 0.06	1.02
AM/PVP = 1:1	0.43 ± 0.3	7.17 ± 1.26	16.67
AM/PVP = 1:4	0.43 ± 0.3	11.59 ± 2.11	26.95
AM/PVP = 1:10	0.43 ± 0.3	29.51 ± 4.22	68.63
AM/Eudragit = 1:1	0.43 ± 0.3	10.77 ± 1.26	24.48
AM/Eudragit = 1:4	0.43 ± 0.3	15.24 ± 2.17	35.44
AM/Eudragit = 1:10	0.43 ± 0.3	38.28 ± 3.21	90.23

## Data Availability

Not applicable.

## Supplementary Materials

The following are available online at https://www.mdpi.com/article/10.3390/polym15143034/s1, Figure S1: The PXRD pattern of AM-PVP 1:10 after 30 days of storage at 25 °C with 0% and 75% RH

## References

[1] Dahan A., Miller J.M., Amidon G.L. (2009). Prediction of Solubility and Permeability Class Membership: Provisional BCS Classification of the World’s Top Oral Drugs. AAPS J..

[2] Ku M.S., Dulin W. (2012). A biopharmaceutical classification-based Right-First-Time formulation approach to reduce human pharmacokinetic variability and project cycle time from First-In-Human to clinical Proof-Of-Concept. Pharm. Dev. Technol..

[3] Okada H., Ueda K., Yasuda Y., Higashi K., Inoue M., Ito M., Noguchi S., Kawakami K., Moribe K. (2020). Correlation between drug dissolution and resistance to water-induced phase separation in solid dispersion formulations revealed by solid-state NMR spectroscopy. Int. J. Pharm..

[4] Takagi T., Ramachandran C., Bermejo M., Yamashita S., Yu L.X., Amidon G.L. (2006). A Provisional Biopharmaceutical Classification of the Top 200 Oral Drug Products in the United States, Great Britain, Spain, and Japan. Mol. Pharm..

[5] Amidon G.L., Lennernäs H., Shah V.P., Crison J.R. (1995). A Theoretical Basis for a Biopharmaceutic Drug Classification: The Correlation of In Vitro Drug Product Dissolution and In Vivo Bioavailability. Pharm Res..

[6] Rosenberger J., Butler J., Dressman J. (2018). A Refined Developability Classification System. J. Pharm. Sci..

[7] Brough C., Williams R.O. (2013). Amorphous solid dispersions and nano-crystal technologies for poorly water-soluble drug delivery. Int. J. Pharm..

[8] Zhao Z., Katai H., Higashi K., Ueda K., Kawakami K., Moribe K. (2019). Cryo-TEM and AFM Observation of the Time-Dependent Evolution of Amorphous Probucol Nanoparticles Formed by the Aqueous Dispersion of Ternary Solid Dispersions. Mol. Pharm..

[9] Budiman A., Higashi K., Ueda K., Moribe K. (2021). Effect of drug-coformer interactions on drug dissolution from a coamorphous in mesoporous silica. Int. J. Pharm..

[10] Bi Y., Xiao D., Ren S., Bi S., Wang J., Li F. (2017). The Binary System of Ibuprofen-Nicotinamide Under Nanoscale Confinement: From Cocrystal to Coamorphous State. J. Pharm. Sci..

[11] Hancock B.C., Zografi G. (1997). Characteristics and Significance of the Amorphous State in Pharmaceutical Systems. J. Pharm. Sci..

[12] Yu L. (2001). Amorphous pharmaceutical solids: Preparation, characterization and stabilization. Adv. Drug Deliv. Rev..

[13] Brouwers J., Brewster M.E., Augustijns P. (2009). Supersaturating Drug Delivery Systems: The Answer to Solubility-Limited Oral Bioavailability?. J. Pharm. Sci..

[14] Chauhan R., Tripathi A., Srivastava K.K. (2014). High-energy ion treatments of amorphous As40Se60 thin films for optical applications. Prog. Nat. Sci. Mater. Int..

[15] Frank D.S., Matzger A.J. (2018). Probing the Interplay between Amorphous Solid Dispersion Stability and Polymer Functionality. Mol. Pharm..

[16] Mistry P., Suryanarayanan R. (2016). Strength of Drug–Polymer Interactions: Implications for Crystallization in Dispersions. Cryst. Growth Des..

[17] Ueda K., Okada H., Zhao Z., Higashi K., Moribe K. (2020). Application of solid-state 13C relaxation time to prediction of the recrystallization inhibition strength of polymers on amorphous felodipine at low polymer loading. Int. J. Pharm..

[18] Pandi P., Bulusu R., Kommineni N., Khan W., Singh M. (2020). Amorphous solid dispersions: An update for preparation, characterization, mechanism on bioavailability, stability, regulatory considerations and marketed products. Int. J. Pharm..

[19] Ilevbare G.A., Liu H., Edgar K.J., Taylor L.S. (2013). Maintaining Supersaturation in Aqueous Drug Solutions: Impact of Different Polymers on Induction Times. Cryst. Growth Des..

[20] Lehmkemper K., Kyeremateng S.O., Heinzerling O., Degenhardt M., Sadowski G. (2017). Long-Term Physical Stability of PVP- and PVPVA-Amorphous Solid Dispersions. Mol. Pharm..

[21] Xie T., Taylor L.S. (2016). Dissolution Performance of High Drug Loading Celecoxib Amorphous Solid Dispersions Formulated with Polymer Combinations. Pharm. Res..

[22] Qin Y., Xiao C., Li X., Huang J., Si L., Sun M. (2022). Enteric Polymer–Based Amorphous Solid Dispersions Enhance Oral Absorption of the Weakly Basic Drug Nintedanib via Stabilization of Supersaturation. Pharmaceutics..

[23] Fan N., Ma P., Wang X., Li C., Zhang X., Zhang K., He Z. (2018). Storage Stability and Solubilization Ability of HPMC in Curcumin Amorphous Solid Dispersions Formulated by Eudragit E100. Carbohydr. Polym..

[24] Wang X., Zhu Y., Zhao X., Zhang S., Cao M., Wang X., Li W. (2022). Development and characterization of an amorphous curcumin-Eudragit® E100 solid dispersions with improved solubility, stability, and pharmacokinetic properties. Pharm. Dev. Technol..

[25] Dening T.J., Taylor L.S. (2018). Supersaturation Potential of Ordered Mesoporous Silica Delivery Systems. Part 1: Dissolution Performance and Drug Membrane Transport Rates. Mol. Pharm..

[26] Greenspan L. (1977). Humidity fixed points of binary saturated aqueous solutions. J. Res. Natl. Bur. Stand. Sect. A Phys. Chem..

[27] Baird J.A., Van Eerdenbrugh B., Taylor L.S. (2010). A Classification System to Assess the Crystallization Tendency of Organic Molecules from Undercooled Melts. J. Pharm. Sci..

[28] Saal W., Ross A., Wyttenbach N., Alsenz J., Kuentz M. (2017). A Systematic Study of Molecular Interactions of Anionic Drugs with a Dimethylaminoethyl Methacrylate Copolymer Regarding Solubility Enhancement. Mol. Pharm..

[29] Indulkar A.S., Mo H., Gao Y., Raina S.A., Zhang G.G.Z., Taylor L.S. (2017). Impact of Micellar Surfactant on Supersaturation and Insight into Solubilization Mechanisms in Supersaturated Solutions of Atazanavir. Pharm. Res..

[30] Lu J., Ormes J.D., Lowinger M., Xu W., Mitra A., Mann A.K.P., Litster J.D., Taylor L.S. (2017). Impact of Endogenous Bile Salts on the Thermodynamics of Supersaturated Active Pharmaceutical Ingredient Solutions. Cryst. Growth Des..

[31] Abdul-Fattah A.M., Bhargava H.N. (2002). Preparation and in vitro evaluation of solid dispersions of halofantrine. Int. J. Pharm..

[32] Sethia S., Squillante E. (2004). Solid dispersion of carbamazepine in PVP K30 by conventional solvent evaporation and supercritical methods. Int. J. Pharm..

[33] Budiman A., Citraloka Z.G., Muchtaridi M., Sriwidodo S., Aulifa D.L., Rusdin A. (2022). Inhibition of Crystal Nucleation and Growth in Aqueous Drug Solutions: Impact of Different Polymers on the Supersaturation Profiles of Amorphous Drugs—The Case of Alpha-Mangostin. Pharmaceutics.

[34] Chiou W.L., Riegelman S. (1970). Oral Absorption of Griseofulvin in Dogs: Increased Absorption via Solid Dispersion—In Polyethylene Glycol 6000. J. Pharm. Sci..

[35] Hancok B.C., Parks M. (2000). What is the true solubility advantage for amorphous pharmaceuticals?. Pharm Res..

[36] Aisha A.F.A., Ismail Z., Abu-salah K.M., Majid A.M.S.A. (2012). Solid dispersions of α-mangostin improve its aqueous solubility through self-assembly of nanomicelles. J. Pharm. Sci..

[37] Choi M.-J., Woo M.R., Choi H.-G., Jin S.G. (2022). Effects of Polymers on the Drug Solubility and Dissolution Enhancement of Poorly Water-Soluble Rivaroxaban. Int. J. Mol. Sci..

[38] Schittny A., Huwyler J., Puchkov M. (2020). Mechanisms of increased bioavailability through amorphous solid dispersions: A review. Drug Deliv..

[39] Budiman A., Aulifa D.L. (2022). A Comparative Study of the Pharmaceutical Properties between Amorphous Drugs Loaded-Mesoporous Silica and Pure Amorphous Drugs Prepared by Solvent Evaporation. Pharmaceuticals.

