# Supplementary figures and images for: Effect of Drug–Polymer Interaction in Amorphous Solid Dispersion on the Physical Stability and Dissolution of Drugs: The Case of Alpha-Mangostin
Source: Polymers (Basel). 2023 Jul 13;15(14):3034. doi: 10.3390/polym15143034 (PMC10384849; doi:10.3390/polym15143034)

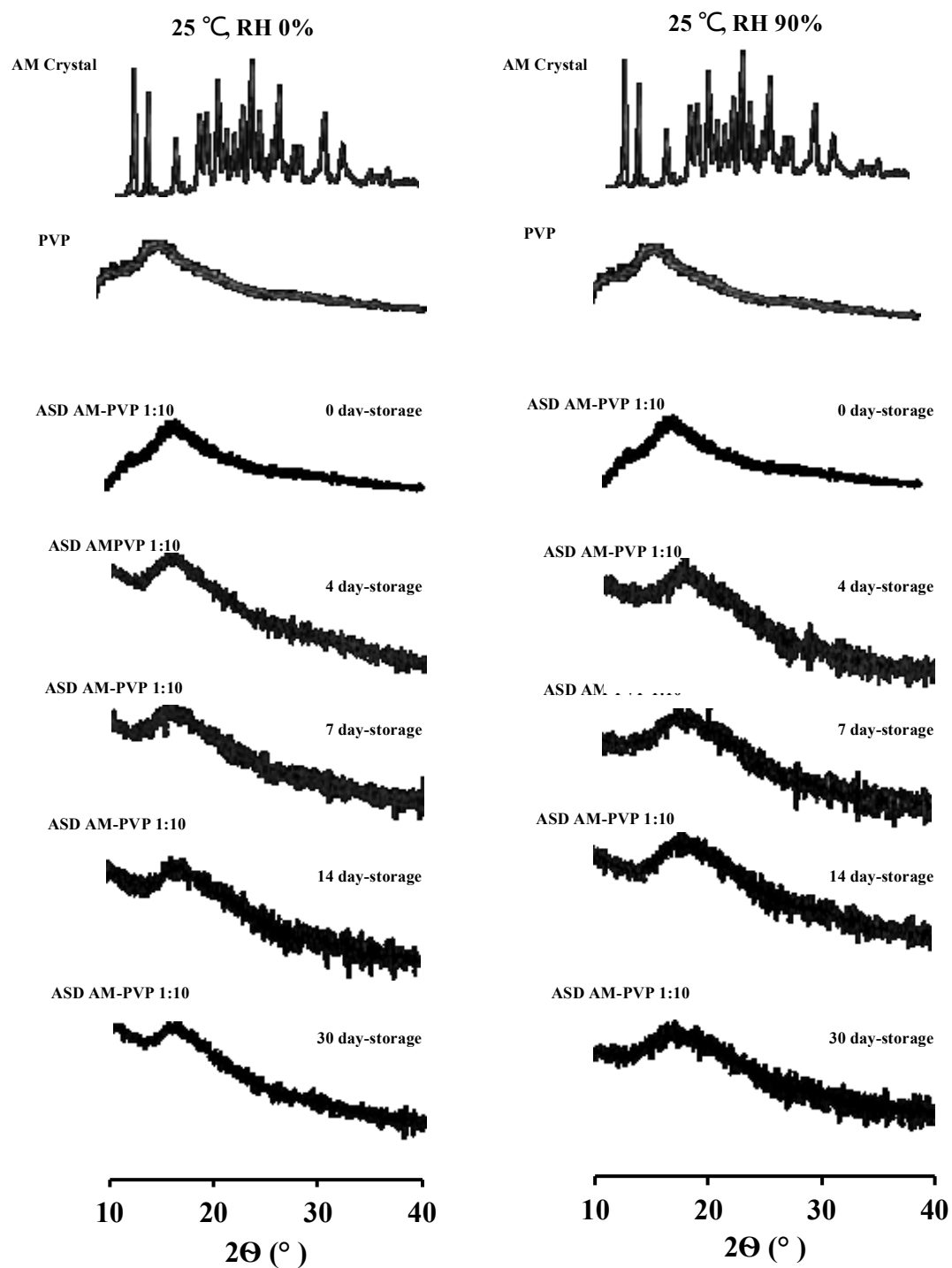

**Figure S1:** The PXRD pattern of AM-PVP 1:10 after 30 days of storage at 25 °C with 0% and 75% RH

Supplement: Supplementary file 1 [file polymers-15-03034-s001.zip › polymers-2485469-supplementary.pdf]
